# Therapy of Pediatric-Onset Multiple Sclerosis: State of the Art, Challenges, and Opportunities

**DOI:** 10.3389/fneur.2021.676095

**Published:** 2021-05-17

**Authors:** Monica Margoni, Francesca Rinaldi, Paola Perini, Paolo Gallo

**Affiliations:** ^1^Multiple Sclerosis Centre of the Veneto Region (CeSMuV), University Hospital of Padua, Padua, Italy; ^2^Padova Neuroscience Centre, University of Padua, Padua, Italy; ^3^Department of Neurosciences, Medical School, University of Padua, Padua, Italy

**Keywords:** pediatric onset multiple sclerosis, first-line therapies, second-line therapies, efficacy, safety

## Abstract

Treatment of pediatric-onset multiple sclerosis (POMS) has been tailored after observational studies and data obtained from clinical trials in adult-onset multiple sclerosis (AOMS) patients. There are an increasing number of new therapeutic agents for AOMS, and many will be formally studied for use also in POMS. However, there are important efficacy and safety concerns regarding the use of these therapies in children and young adults. This review will discuss the current state of the art of POMS therapy and will focus on the newer therapies (oral and infusion disease-modifying drugs) and on those still currently under investigation.

## Introduction

High relapse rate, rapid accumulation of white (WM) and gray matter (GM) damage, and worse long-term physical and cognitive disability are typical features of pediatric-onset multiple sclerosis (POMS) (1–6). Despite neuroplasticity, POMS patients reach similar levels of disability at a younger age than adult-onset MS (AOMS), and their quality of life (QoL) is frequently significantly compromised, with negative impacts on school, social, and physical activities (5, 7). Accordingly, POMS has to be considered a severe, highly disabling disease, with extremely high social costs. Approximately, POMS accounts for 2–10% of all MS cases (5), but incidence of MS in children and adolescents is increasing, and it has become relatively frequent to face the diagnosis and the treatment of this peculiar population.

Since no definite guideline exists on the management of POMS, treatment strategies often reflect the center-specific experience as well as the neurologist's therapeutic attitude and knowledge that derive from the application of adult-tailored MS therapeutic protocols. Despite heterogeneity, data on efficacy and safety of disease-modifying drugs (DMDs) [e.g., interferon beta (IFN β), glatiramer acetate (GA), natalizumab (NTZ), and rituximab] in POMS collected from single- of multi-center open-label observational studies indicate a marked effect on clinical and magnetic resonance imaging (MRI) parameters of inflammation (8–11), especially when therapy is initiated very early (12), as also pointed out by the 2012 International Pediatric MS Study Group (IPMSSG) consensus (13).

Recently, the US Network of Pediatric MS Centers reported data on 741 POMS patients, 197 treated with newer therapies [fingolimod, dimethyl fumarate (DMF), teriflunomide, NTZ, rituximab, and ocrelizumab] and 544 treated with IFN β or GA. As expected, those on newer DMDs had significant lower annualized relapse rate (ARR) than those with IFN β or GA (*p* < 0.001) (14). Moreover, a high rate of IFN β and GA treatment failure has been reported in POMS, ranging from 25 to 64% across studies (15). It is noteworthy that many of these drugs are still used off-label; thus, the recent approval of fingolimod for POMS by the Food and Drug Administration (FDA) and European Medicines Agency (EMA) constitutes a significant step forward in treating these patients (16).

Finally, the QoL must be strongly taken into consideration when treating POMS with DMDs. Therapies that induce symptoms (fever, headache, myalgias, etc.) that negatively and persistently impact school and sport performances, and therefore substantially modify the QoL, must be avoided or interrupted early.

Here, we review the state of the art of POMS therapy and focus on the newer therapies (oral and infusion DMDs) and on those currently under trial.

## First-Line Therapies

### Injectables

IFN β and GA (hereafter called injectables) are the most widely used DMDs in POMS (17–19). Both drugs showed a high-efficacy profile in the short term (see Table 1 for a comprehensive overview) (18, 20–23, 28, 29) but also a consistent rate of treatment failure in the medium/long term. The US and Italian Network of the MS Centers collected longitudinal data on injectable-treated POMS, summarized in two reports: (1) after a mean follow-up of 3.9 years, 114 (44.2%) of 258 patients had their therapy changed to a second DMDs owing to refractory disease (27.9%) or poor tolerability of a first-line DMDs (16.3%) (30); (2) after a follow-up of 12.5 years, 82/97 (84.5%) patients needed a therapy switch, that in up to 58% of cases, was an immunosuppressive/second-line drug (9).

**Table 1:** Observational and clinical studies on first-line immunomodulatory therapies in pediatric multiple sclerosis.

**Treatment**	**First author**	**Year**	**Trial design**	**Number of patients**	**Clinical findings**	**MRI findings**	**Adverse effects**
IFN β	Mikaeloff et al. (20)	2001	Prospective study (median FU 1 year)	16 RRMS	Stable EDSS	Stable T2 lesions in 3 patients ↑ T2 lesions in 6 patients	Fever: 50% Headache: 28% Myalgia: 17% Fatigue: 5%
	Ghezzi et al. (21)	2005	Prospective study (mean FU 34.4 ± 25.0 months in Rebif-Betaferon, 23.3 ± 13.4 months in Avonex)	18 RRMS Rebif-Betaferon 38 RRMS Avonex	↓ ARR (3.29 ± 2.3 at b vs. 0.86 ± 0.8 at FU in Rebif-Betaferon) ↓ ARR (2.49 ± 1.4 at b vs. 0.49 ± 0.5 at FU in Avonex)	–	<10% of patients
	Banwell et al. (19)	2006	Retrospective study (mean FU 29.2 months)	43 RRMS	–	–	Flu-like syndrome (35%), abnormal liver function test (26%), and injection site reaction (21%) No SAE
	Tenambaum et al. (18)	2006	Open-label, prospective, single-center study (6 years)	24 RRMS	↓ ARR (1.7 at b vs. 0.04 at y5)	–	96% of patients (58% flu-like syndrome, 17% myalgia/arthralgia)
	Mikaeloff et al. (22)	2008	Prospective study (mean FU 5.5 years)	197 RRMS	↓ Rate of the first attack during the first year of treatment (hazard ratio: 0.31, 95% confidence interval: 0.13–0.72) and 2 years (0.40, 0.20–0.83)	–	–
GA	Kornek et al. (23)	2003	Prospective study (24 months)	7 RRMS	2/7 relapse free EDSS stable	↓T2 lesions in 2/7 ↑ T2 lesions in 3/7	–
	Ghezzi et al. (21)	2005	Prospective study (mean FU 33.3 ± 27.6 months)	9 RRMS	↓ ARR (2.89 ± 1.3 at b vs. 0.26 ± 0.36 at FU)	–	–
Dimethyl fumarate	Makhani et al. (24)	2016	Retrospective study (median FU 15 months)	13 RRMS	↓ ARR in 8/13 children	New T2 lesions in 33%, one of whom had been non-adherent to treatment	8/13 (62%) flushing 7/13 (54%) GI discomfort, 3/13 rash (23%), 2/13 malaise (15%)
	Alroughani et al. (25)	2018	Phase II, single arm, multicenter, open label (FOCUS) (24 weeks)	22 RRMS	↓ ARR (1.5 at b vs. 0.8 at 24 weeks)	↓ New/enlarged T2 hyperintense lesions (−1.5 at b vs. −8.0 at 24 weeks, p = 0.009)	73% of patients (abdominal pain, nausea, vomiting, flushing) No SAE
	Alroughani et al. (26)	2020	Extension study of FOCUS (CONNECTED) (96 weeks)	20 RRMS	↓ ARR (1.5 at b vs. 0.2 at 120 weeks, *p* < 0.0001)	12/17 (71%) no new/enlarged T2 hyperintense lesions from w16 to w24	90% AEs (flushing in 25%) No SAE
Teriflunomide	Chitnis et al. (27)	2020	Double-blind, randomized, placebo-controlled Phase III (TERIKIDS) (96 weeks)	109 RRMS	↓ Clinical relapse by 34% (*p* = 0.29) ↓ Time of relapse or switch due to high MRI activity by 43% (*p* = 0.041)	↓ Gd+ and new/enlarged T2 hyperintense lesions (1.9 vs. 7.5, *p* < 0.0001 and 4.7 vs. 10.5, *p* = 0.0006, respectively) compared with Pbo	88.1% AEs 3 SAEs (pulmonary tuberculosis, acute pancreatitis, ALT increase)
	Chitnis et al. (27)	2020	Open label extension (TERIKIDS) (96 weeks)	100 RRMS	↓ First confirmed relapse and 24-week sustained disability progression compared with Pbo/Ter (46.0 vs. 64.0% and 17.4 vs. 29.3%).	↓ Gd+ and new/enlarged T2 hyperintense lesions in the Ter-treated group (1.9 vs. 4.2, *p* = 0.0106, 6.3 vs. 13.0, *p* = 0.0006)	↑AEs in the Pbo/Ter compared with the Ter/Ter (82.7% vs. 68.0%) 2 SAEs (acute pancreatitis, amylase and lipase increased)

*B, baseline; EDSS, Expanded Disability Status Scale; FU, follow-up; GA, glatiramer acetate; IFN β, interferon β; Pbo, placebo; RR, relapse rate; RRMS, relapsing–remitting multiple sclerosis; Ter, teriflunomide*.

The US Network of Pediatric MS Centers analyzed 618 DMD-treated patients and reported that 147/483 (30.4%) of those treated with injectables switched to other therapies in a mean follow-up of 3.5 years (17). More recently, in a cohort of 741 patients, the 197 who were commenced on newer therapies (DMF, fingolimod, teriflunomide, NTZ, rituximab, and ocrelizumab) had significantly lower ARR than the 544 on injectable, confirming the higher efficacy of the newer therapies (14).

Although injectables are not associated with increased risk of infections or malignancies and the most reported side effects are injection site reactions for GA and flu-like symptoms for IFN β (25–35%) (19, 31), the loss of adherence (i.e., missing > 20% of doses) is high and is more frequently reported by patients (up to 41%) than by parents (14%) or pharmacist (7%) (15). Expert opinion suggests that IFN β is better tolerated if initiated at 25–50% of the standard dose followed by a gradual escalation to full dose over 1 to 3 months (32).

### Orals

#### Dimethyl Fumarate

A phase II multicenter study (FOCUS) (25) with DMF (120 mg twice daily on days 1–7, 240 mg twice a day thereafter) on 22 POMS (20 of which completed the study) showed a median change in number of new/enlarged T2 hyperintense lesions of −2.0 at week 24 compared with baseline (−1.5 vs. −8.0, *p* = 0.009). The unadjusted ARR was 1.5 in the year prior to study and 0.8 at week 24. Adverse events (AEs) (most commonly gastrointestinal disorders and flushing) and pharmacokinetic (PK) were consistent with those observed in adults (25). The good safety and tolerability profiles of DMF were confirmed, in agreement with a previous small retrospective study on nine patients (24).

In the CONNECTED study (26), extension of FOCUS, a long-lasting benefit of therapy, was observed: 12/17 participants (71%) had no new/enlarged T2 hyperintense lesions from weeks 16 to 24. Over a mean treatment period of 120 weeks, a significant reduction of ARR compared with the year before DMF initiation was observed (from 1.5 to 0.2, *p* < 0.0001). AEs were reported in 18 patients (90%) during the 24-week follow-up (the most frequent being flushing, observed in 25% of the patients). However, no patient experienced severe AEs (SAEs) leading to DMF discontinuation.

A phase III, double-blind, placebo-controlled, three-arm randomized controlled trial (RCT) aiming on evaluating safety and efficacy of DMF compared with placebo and pegylated IFN β-1a is currently recruiting patients (ClinicalTrials.gov Identifier: NCT03870763).

#### Teriflunomide

The results of a 96-week, double-blind, randomized, placebo-controlled phase III RCT evaluating efficacy, safety, and PK of teriflunomide in 109 POMS aged 10–17 years (TERIKIDS) have been presented at ECTRIMS 2020 (27).

Teriflunomide numerically reduced the risk of clinical relapse by 34% relative to placebo, but this did not reach statistical significance (*p* = 0.29). Conversely, teriflunomide reduced the risk of the time of clinical relapse or switch due to high MRI activity by 43% (*p* = 0.041) and the appearance of Gd-enhancing and new/enlarged T2 hyperintense lesions compared with placebo (1.9 vs. 7.5, *p* < 0.0001 and 4.7 vs. 10.5, *p* = 0.0006, respectively). Three SAEs were observed [pulmonary tuberculosis, acute pancreatitis, and alanine aminotransferase (ALT) increase].

The open-label extension of this RCT is currently in progress. An interim analysis on 100 patients demonstrated that the time to first confirmed relapse and the 24-week sustained disability progression were numerically lower for the teriflunomide/teriflunomide (T/T) arm compared with the placebo/teriflunomide (P/T) arm but did not reach the significance (46.0 vs. 64.0%, *p* = 0.098 and 17.4 vs. 29.3%, respectively).

Furthermore, new/enlarged T2 hyperintense and Gd-enhancing lesions were significantly reduced in the T/T arm (6.3 vs. 13.0, *p* = 0.0006, and 1.9 vs. 4.2, *p* = 0.0106, respectively). The incidence of AEs was higher in the P/T arm compared with the T/T arm during the open-label period (82.7 vs. 68.0%, respectively). Two SAEs were recorded (acute pancreatitis; increased amylase and lipase). Although teriflunomide proved to be well-tolerated and disclosed a manageable safety profile, the SAEs mentioned above suggest that if this medication is ultimately approved, an adequate surveillance of biological parameters in treated patients is necessary. Finally, the placebo-controlled design for TERIKIDS raised some concerns on its inherent limiting ability to compare clinical trials with one another (33). Further studies with an active comparator and other MRI endpoints (e.g., annualized rate of brain atrophy) are warranted to further define the efficacy and safety profile of teriflunomide.

## Second-Line Therapies

### Orals

#### Fingolimod

The randomized, double-blind, phase III RCT PARADIG*MS* (34) compared fingolimod with intramuscular (i.m.) IFN β-1a in a cohort of 215 patients (34). This study demonstrated a significant reduction of the adjusted ARR in the 107 patients treated with fingolimod compared with those treated with IFN β-1a (0.12 vs. 0.67, *p* < 0.001). Furthermore, new/enlarged T2 hyperintense lesions were reduced in fingolimod patients compared with IFN β-1a (4.39 vs. 9.27, *p* < 0.001). AEs occurred in 88.8% of patients who received fingolimod and in 95.3% of those who received IFN β-1a. SAEs occurred in 18 patients (16.8%) in the fingolimod group and included four cases of infections (appendicitis, cellulitis, gastrointestinal infection, oral abscess, viral infection, and viral pharyngitis) and six (5.6%) cases of convulsions [vs. 1 (0.9%) in the IFN β-1a arm]. Other SAEs in the fingolimod group included single cases (0.9%) of agranulocytosis, arthralgia, autoimmune uveitis, bladder spasm, dyspepsia, dysuria, elevated alanine aminotransferase level, elevated γ-glutamyl transferase level, gastrointestinal necrosis (intussusception or necrotic bowel), head injury, humerus fracture, hypersensitivity vasculitis, migraine, migraine without aura, muscular weakness, rectal tenesmus, second-degree atrioventricular block, and small-intestinal obstruction and two cases of leukopenia (1.9%).

In a secondary analysis on MRI parameters (16), fingolimod demonstrated a reduction in the annualized rate of formation of new/enlarged T2 hyperintense lesions (52.6%, *p* < 0.001), number and annualized rate of T1 hypointense lesions (66% and *p* < 0.001; 62.8% and *p* < 0.001, respectively), and combined unique active lesions (60.7%, *p* < 0.001) vs. IFN β-1a. Furthermore, the percent increase in T2 (18.4 vs. 32.4%, *p* < 0.001) and Gd-enhancing T1 lesion (−72.3 vs. 4.9%, *p* < 0.001) volumes and the annualized rate of brain atrophy (−0.48 vs. −0.80%, *p* = 0.014) were lower with fingolimod vs. IFN β-1a.

Prior to PARADIG*MS*, data from two small observational studies were available. In a study on 23 highly active POMS patients treated with fingolimod (35), a significant decrease in ARR (75%), new T2 hyperintense (81%), and Gd-enhancing (93%) lesions compared with pretreatment was reported. Noteworthy, seven patients with very high disease activity at clinical presentation experienced disease re-activation when switching from NTZ to fingolimod after a 2-month washout period. Six of them were further switched to alemtuzumab during the follow-up. These data suggested that very active POMS does not probably respond to fingolimod and needs to be treated with more potent immunosuppressive drugs. No SAE was reported in this study. In a second study conducted on 17 POMS treated with fingolimod for an average of 8.6 months, the majority of the patients remained free of clinical or radiological activity. An improvement in Expanded Disability Status Scale (EDSS) score compared with pretreatment was also observed (range of change −3 to −0.5) (36).

All together, these observations suggest fingolimod to be effective and well-tolerated in most POMS.

#### Cladribine

No data are currently available on cladribine-treated POMS.

### Infusion Therapies

#### Natalizumab

Following the approval of fingolimod, in Italy, NTZ has been approved for POMS aged 12–17, having active and rapidly evolving MS not responsive to fingolimod, or in the presence of contraindications or persistent side effects due to fingolimod. No RCT has been conducted on POMS to date, but several observational studies have focused on NTZ efficacy and safety in these patients (see Table 2 for a comprehensive overview).

**Table 2:** Observational and clinical studies on second-line immunomodulatory therapies in pediatric multiple sclerosis.

**Treatment**	**First author**	**Year**	**Trial design**	**Number of patients**	**Clinical findings**	**MRI findings**	**Adverse effects**
Natalizumab	Kornek et al. (37)	2013	Retrospective study (mean FU 11 months)	20 RRMS	↓ARR (3.7 at b vs. 0.04, *p* < 0.001) ↓ EDSS (2 at b vs. 1; *p* < 0.02)	↓ T2 lesions (7.8 at b vs. 0.5; *p* < 0.001)	50% (headache, asthenia, infections, and hypersensitivity)
	Arnal-Garcia et al. (38)	2013	Retrospective study (mean FU 17 months)	8 RRMS	↓ARR (3 at b vs. 0) ↓ EDSS (3 at b vs. 1)	No Gd+ lesion at FU	3 AEs
	Ghezzi et al. (39)	2013	Retrospective study (mean FU 26 months)	55 RRMS	3 relapses ↓ EDSS (2.7 at b vs. 1.9, *p* < 0.001)	88% free from radiological disease	Transitory AEs in 22/55 patients (headache, upper respiratory disorders, vertigo)
	Ghezzi et al. (40)	2015	Retrospective study (mean FU 26 months)	101 RRMS	↓ARR (2.3 ± 1.0 at b vs. 0.1 ± 0.3, *p* < 0.001) ↓ EDSS (2.6 ± 1.3 at b vs. 1.8 ± 1.2, *p* < 0.001)	T2/Gd+ lesions were observed in 10/91 (10.9 %) patients at 6 months, 6/87 (6.9 %) at 12 months, 2/61 (3.3 %) at 18 months, 2/68 (2.9 %) at 24 months, 3/62 (4.8 %) after 30 months	AEs in 36/101 (headache, upper respiratory disorders, vertigo)
	Margoni et al. (41)	2020	Retrospective study (24 months)	20 RRMS	↓ EDSS (2.6 ± 0.7 at b vs. 1.5 ± 0.5, *p* < 0.0001)	2 patients new T2 lesions	No AE
Alemtuzumab	Margoni et al. (42)	2019	Case series (mean FU 3.9 years)	5 RRMS	No relapse 3 patients had clinical improvement	No MRI activity	No SAE
	Jure Hunt et al. (43)	2020	Case series	2 RRMS	No relapse EDSS improvement	No MRI activity	No SAE
Rituximab	Dale et al. (44)	2014	Multicenter retrospective study (mean FU 3.3)	4 RRMS	Benefit: 1 definite, 0 probable, 1 possible, 1 none, 1 worsening	–	12.5% AEs (anaphylaxis in 3, 11 7.6% infections, 2 deaths)
	Salzer et al. (45)	2016	Retrospective study (median FU 23.6 months)	14 RRMS	EDSS stable in 93% of patients	1 lesion detected on MRI	No AE
Cyclophosphamide	Makhani et al. (46)	2009	Retrospective study (mean FU 2.7 years)		↓ARR (from 3.8 to 1.1, >70%) ↓ or stable EDSS in 83%	↓ T2 and gad+ lesions (>75%)	Nausea and vomiting: 88%
Fingolimod	Chitnis et al. (34)	2018	Randomized, double-blind, phase III trial (PARADIG*MS*) (24 months)	215 RRMS	↓ ARR (0.12 FTY vs. 0.67 IFN β, *p* < 0.001)	↓ T2 lesions (4.39 FTY vs. 9.27 IFN β, *p* < 0.001)	SAEs in 16.8% (infection, leukopenia, 6 patients had convulsions)
	Huppke et al. (35)	2019	Retrospective study (mean FU 31 months)	23 RRMS	↓ 75% ARR	↓ 81% T2 lesions	-
	Arnold et al. (16)	2020	Randomized, double-blind, phase III trial (PARADIG*MS*) (24 months)	215 RRMS	–	↓52.6% T2 lesions in FTY vs. IFN β (*p* < 0.001) ↓ 66% T1 lesions in FTY vs. IFN β (*p* < 0.001)	–

*B, baseline; EDSS, Expanded Disability Status Scale; FTY, fingolimod; FU, follow-up; IFN β, interferon β; Pbo, placebo; RR, relapse rate*.

The largest cohort of NTZ-treated POMS included 101 patients, having a mean age at onset of 12.9 ± 2.7 years and a mean EDSS of 2.6. Sixty-six percent had been previously treated with first-line DMDs. Patients were treated with NTZ for a mean period of 34.2 ± 18.3 months (40). Compared with baseline, a significant reduction in the mean ARR (from 2.3 ± 1.3 to 0.1 ± 0.3, *p* < 0.001) and new Gd-enhancing lesions (82.8 vs. 10.6%, *p* < 0.001) were observed at the end of the follow-up (40). Moreover, the no evidence of disease activity (NEDA)-3 status (i.e., no clinical relapses, no increase in disability, and no MRI activity) was achieved in 58% patients. These observations were confirmed in other observational studies (37–39, 41, 47). Recently, we studied the achievement of the NEDA-3 plus status, which includes cognition in the NEDA-3 (the cognitive decline was defined as a decrease of at least four points in the Symbol Digit Modality Test), in 20 naïve POMS treated with NTZ. We observed that 17/20 (85%) and 18/20 (80%) of patients achieved NEDA-3 plus at months 12 and 24, respectively (41).

NTZ was found to be well-tolerated and safe in POMS patients. In some studies, no clinical AE was experienced (39, 47). An open-label, multiple-dose, multicenter prospective study aimed to evaluate the PK/pharmacodynamic (PK/PD) profile, safety, and tolerability of NTZ in POMS, aged 10–18 years, demonstrated similar profiles in adults and pediatric patients in the short term (48). Longer studies, also including a larger number of younger subjects (aged 10–12 years), are required to further inform about long-term PK and PD parameters in POMS. Recently, some concerns about immunosuppression in MS were raised during the coronavirus disease 2019 (COVID-19) pandemic. In our experience, NTZ did not expose POMS to a higher risk of SARS-CoV-2 infection or to a clinically overt/severe disease (49).

At present, no cases of progressive multifocal leukoencephalopathy (PML) have been reported in POMS patients treated with NTZ. The prevalence of JCV seropositivity in POMS was reported to be higher [ranging from 39 to 51.6% (39, 40, 50)] than in the general healthy pediatric population [21% (51)] but lower than in AOMS patients (52).

While tolerability and safety data are reassuring and clearly indicated that NTZ can be considered the treatment of choice for very active POMS, long-term safety data on larger cohort of patients are needed, especially for evaluating the risk of PML.

#### Anti-CD20 Therapies: Rituximab and Ocrelizumab

The first anti-CD20 monoclonal antibody (MAb) used in MS is the chimeric antibody rituximab, currently prescribed off-label in case of a highly active disease. In POMS, rituximab is one of the most used second-line immunosuppressive therapies.

In a case series of 14 POMS treated with rituximab for a median period of 23.6 months, a stable disease was observed in 13/14 patients (93%) with no SAE reported (45). However, in a cohort of 144 pediatric patients with autoimmune and inflammatory central nervous system (CNS) disorders (among which four POMS), infusion AEs were recorded in 18/144 (12.5%), including grade 4 (anaphylaxis) in three; 11 patients (7.6%) had infections, including two with grade 5 (death) and two with grade 4 (disabling) (44). Furthermore, in a cohort of 1,019 pediatric patients with MS and clinically isolated syndromes, side effects and tolerability were similar to those reported in adults (14, 17). No rituximab-related PML cases have been reported in POMS. In the position paper of the International Pediatric MS Study Group, the potential benefit of rituximab was highlighted, but the need for a better evaluation of the optimal dosing, and the safety and efficacy profile were also stressed (13, 53).

Currently, there are no published reports of ocrelizumab use in POMS. An RCT evaluating the PK/PD and the efficacy of ocrelizumab 600 mg i.v. (300 mg i.v. if body weight <40 kg) in POMS is in progress (https://clinicaltrials.gov/ct2/show/NCT04075266).

#### Alemtuzumab

A phase III RCT aimed to evaluate safety and efficacy of alemtuzumab in POMS patients who have failed at least two DMDs is in progress (NCT03368664). Some observational reports showed that alemtuzumab was relatively well-tolerated and effective in POMS; indeed, no serious infusion reactions, infections, or relapses were recorded during the follow-up (42, 43).

#### Cyclophosphamide

Several studies have suggested that cyclophosphamide treatment may be most beneficial in younger adult MS patients (54–56). A single, multicenter retrospective study of 17 cyclophosphamide-treated POMS with a mean follow-up of 2.7 years showed a reduction in ARR (from 3.8 to 1.1), and stabilization of disability scores assessed 1 year after treatment initiation in most patients (83%) compared with baseline. Furthermore, a reduction in new/enlarged T2 hyperintense lesions and Gd-enhancing lesions was observed (100 vs. 75% and 91 vs. 67%, respectively) (46). The most frequent AEs included vomiting, transient alopecia, osteoporosis, and amenorrhea. One patient developed bladder carcinoma that was successfully treated (46).

#### Mitoxantrone

Given the risk of cardiotoxicity and acute myeloid leukemia (57), the use of mitoxantrone in POMS is discouraged.

## Future Perspectives and Conclusions

POMS is a rare but severe form of MS, characterized by a more prominent clinical and radiological activity and younger age at reaching cognitive and physical disability milestones, even when treated with first-line DMDs. Furthermore, adherence to injectable DMDs is an important determinant of treatment efficacy in real-world clinical settings. Off-label use of newer DMDs is increasing in POMS and retrospective studies, case series, and phase II trials, indicate that this approach appears to be highly effective and safe in children. However, great efforts should be devoted to design RCTs in POMS. The low number of patients and the potentially severe long-term prognosis strongly indicate the necessity of identifying new and adequately powered MRI targets (e.g., annualized rate of brain atrophy) of treatment as well as more specific clinical (especially cognitive) endpoints for this peculiar MS population. Moreover, the harmonization of regulatory requirements for testing of new treatment should be prioritized to compare clinical trials with one another (33).

Although more data are needed before standardizing the use of first- and second-line newer therapies in POMS, the treatment paradigm implies to design therapeutic strategies based on highly effective drugs. Thus, the approval of fingolimod and the availability of high-efficacy Mab constitute a real step-forward in POMS management.

## Author Contributions

MM, PG, FR, and PP: design and drafting of the manuscript. All authors contributed to the article and approved the submitted version.

## Conflict of Interest

MM reports grants and personal fees from Sanofi Genzyme, Merck Serono, Novartis and Almirall. She was awarded a MAGNIMS-ECTRIMS fellowship in 2020. FR reports grants and personal fees from Genzyme Sanofi, Merck Serono, Biogen Idec, grants from Novartis, during the conduct of the study. PP reports grants and personal fees from Merck Serono, Biogen Idec, Genzyme Sanofi, Roche, Novartis, during the conduct of the study. PG reports grants and personal fees from Merck Serono, Biogen Idec, Genzyme Sanofi, grants and personal fees from Novartis, grants from University of Padua, Department of Neurosciences DNS, grants from Veneto Region of Italy, grants from Italian Association for Multiple Sclerosis (AISM), grants from Italian Ministry of Public Health, during the conduct of the study.
